# Creating and Activating an Implementation Community to Drive HPV Vaccine Uptake in Texas: The Role of an NCI-Designated Cancer Center

**DOI:** 10.3390/vaccines11061128

**Published:** 2023-06-20

**Authors:** Rosalind S. Bello, Michael T. Walsh, Blake Harper, Charles E. Amos, Katherine Oestman, Stephanie Nutt, Marcita Galindez, Kaitlyn Block, Ruth Rechis, Erica M. Bednar, Jennifer Tektiridis, Lewis Foxhall, Mark Moreno, Sanjay Shete, Ernest Hawk

**Affiliations:** 1 The HPV Vaccination Initiative, The University of Texas MD Anderson Cancer Center, Houston, TX 77030, USA; 2 Office of Health Policy, The University of Texas MD Anderson Cancer Center, Houston, TX 77030, USA; 3 Cancer Prevention and Control Platform, The University of Texas MD Anderson Cancer Center, Houston, TX 77030, USA; 4 Impact Evaluation Core, The University of Texas MD Anderson Cancer Center, Houston, TX 77030, USA; 5 Joint Center on Geospatial Analysis & Health, Houston, TX 77030, USA; 6 Division of Cancer Prevention and Population Sciences, The University of Texas MD Anderson Cancer Center, Houston, TX 77030, USA; 7 Government Relations, The University of Texas MD Anderson Cancer Center, Houston, TX 77030, USA; 8 Department of Epidemiology, The University of Texas MD Anderson Cancer Center, Houston, TX 77030, USA

**Keywords:** HPV, HPV vaccination, vaccination, dissemination, implementation, scale, cancer center, cancer prevention, evidence-based interventions, community of practice, implementation network

## Abstract

The University of Texas MD Anderson Cancer Center, a comprehensive cancer center designated by the National Cancer Institute (NCI), defines its service population area as the State of Texas (29.1 M), the second most populous state in the country and the state with the greatest number of uninsured residents in the United States. Consistent with a novel and formal commitment to prevention as part of its core mission, alongside clear opportunities in Texas to drive vaccine uptake, MD Anderson assembled a transdisciplinary team to develop an institutional Framework to increase adolescent HPV vaccination and reduce HPV-related cancer burden. The Framework was developed and activated through a four-phase approach aligned with the NCI Cancer Center Support Grant Community Outreach and Engagement component. MD Anderson identified collaborators through data-driven outreach and constructed a portfolio of collaborative multi-sector initiatives through review processes designed to assess readiness, impact and sustainability. The result is an implementation community of 78 institutions collaboratively implementing 12 initiatives within a shared measurement framework impacting 18 counties. This paper describes a structured and rigorous process to set up the implementation of a multi-year investment in evidence-based strategies to increase HPV vaccination that solves challenges preventing implementation of recommended strategies and to encourage similar initiative replication.

## 1. Introduction

### 1.1. Purpose and Significance

Human papillomavirus (HPV) vaccination can protect against over 90% of cancers caused by HPV, as well as precancers (abnormal cells that can lead to cancer) [1]. A vaccine to prevent HPV-related cancers became available in the United States (U.S.) in 2006. The American Cancer Society reports a 65% reduction in cervical cancer incidence in 2012–2019, which aligns with the first cohort of women to receive the HPV vaccine when they were ages 9–11 [2].

The Centers for Disease Control and Prevention (CDC) recommends routine HPV vaccination of persons aged 11–12 years, however, the vaccination can be given with earliest initiation at 9 years of age. Two doses are recommended for persons initiating the HPV vaccine at ages 9–14 years and three doses if initiating the vaccine at ages 15–26 years [3]. The U.S. average rate for up-to-date HPV vaccination among 13- to 17-year-olds in 2021 was 61.7%, but the rate in Texas was just 51.5%, resulting in Texas ranking 47th of the 50 states in HPV vaccination coverage in 2021. Despite the strong evidence for vaccination and the clear recommendations, both Texas and the U.S. fall short [4,5,6].

While rates of up-to-date vaccination in the U.S. have increased every year since the vaccine was introduced [7], rates in Texas recently declined (2020–2021), as did rates in Houston (2017–2018), San Antonio (2018–2019 and 2020–2021), and rural areas of Texas (2020–2021) [4]. Of the HPV-associated cancer cases (i.e., oropharyngeal, anal, penile, cervical, vulvar, and vaginal) diagnosed among Texans in 2013–2017, 83% of them in females and 74% in of them in males were attributable to HPV. The Texas Cancer Registry reported that approximately 3200 new cases of HPV-associated cancers occurred in Texas each year between 2013 and 2017, with an estimated 80% of the associated cases directly attributed to HPV infection. This represents an estimated 1900 females and 1300 males each year. Of HPV-associated cancers, cervical cancer had the highest incidence among females, with the cervical cancer incidence higher in Texas than rates for the U.S. overall, and oropharyngeal cancer had the highest incidence among males [8].

National Immunization Survey–Teen 2021 (NIS-Teen) reported that lower rates of HPV vaccination and up-to-date rates exist among U.S. adolescents without any health insurance [4]. Texas has the highest rate of uninsured persons in the nation, at almost twice the national average, which includes having the most uninsured children in the country [7]. People without health insurance are less likely to receive prevention and screening services or care to manage chronic disease, which could lead to more costly care in the future.

Vaccines for Children (VFC) is a program focused on helping families who cannot afford vaccination stay on schedule with childhood vaccination as recommended by the Advisory Committee on Immunization Practices (ACIP). VFC is a federally funded program through the Centers for Medicare & Medicaid Services (CMS) with discounted purchasing and distribution to care providers via the CDC. VFC grantees distribute vaccines at no charge [9]. Texas has participated in VFC since 1994 [10]. The program provides vaccines at no cost to providers to immunize children (birth–18 years of age) who meet the eligibility requirements. However, unreimbursed vaccine administration costs are commonly transferred to the adolescents and their caregivers. The lack of reimbursement and appropriate billing codes for vaccine administration combined with infrastructure and administrative paperwork dissuades practices from participating in the VFC program [11].

Under the Affordable Care Act (Patient Protection and Affordable Care Act of 2010), states were provided the opportunity to expand Medicaid to cover a larger portion of their low-income population [12], and the HPV vaccine was included in mandated vaccine coverage (without co-pay) under the preventive health care clause (Patient Protection and Affordable Care Act of 2010). Thirty-seven states, including eight states that are similar in size to Texas and considered Texas’ primary competitors for business and talent, have expanded Medicaid to cover a larger portion of their low-income population [7]. Texas did not expand Medicaid coverage under the Affordable Care Act, despite 5 million Texans (18%) being uninsured [13]. While Texas has approximately 3.6 million children enrolled in Medicaid [14] 11.8% of its children remain uninsured [15]. Of these, uninsured teens, ages 13–17 years, are reported to have the lowest rates of completing recommended adolescent immunizations required to protect them from vaccine preventable diseases [16], as compared to those with private insurance only, any Medicaid, or other insurance.

### 1.2. Problem Statement

Barriers preventing HPV vaccination in Texas are multi-fold and go beyond insurance coverage issues. Patient-level barriers contributing to lower and decreasing HPV vaccination rates in Texas include lack of provider recommendations and lack of information related to HPV risks and vaccines [17]. All vaccines were affected by missed opportunities in the primary care setting during the first year of the pandemic [18]; however, the HPV vaccine has the additional specific challenge that it requires more than one dose to be administered as currently recommended.

Barriers at the provider and system levels include a lack of clinic operational systems that support vaccination, such as optimized electronic health records and standing orders [19]. Recognizing these trends, some clinics have employed evidence-based interventions to address the issue but have found challenges due to changes in delivery of care and logistical issues, such as staff decrease/turnover and competing priorities [5,20].

Primary care clinics are the typical setting for interventions focused on increasing vaccination rates. However, primary care clinics are overburdened and experience time pressures to complete routine visits [21,22]. Despite these environmental constraints, their efforts to increase HPV vaccination have achieved a measure of success [5]. This success may be more modest in geographic areas with fewer resources due to overly large patient populations and insufficiently sized teams (or numbers of providers) to perform current duties [23]. Both clinicians and patients are interested in overcoming access to care problems that can derail intervention efforts [24,25], but the systems-level problems may become increasingly challenging as health care demands are increasing more rapidly than the number of practicing primary care professionals [26,27].

Facilitators to HPV vaccination include application of evidence-based interventions to increase HPV vaccination include implementing standing orders; implementing reminder/recall systems; optimizing electronic health record interoperability with ImmTrac2, Texas’ immunization registry; providing training for and following best practices for provider interventions (e.g., making strong recommendations through use of verbal announcement language, bundling HPV with other vaccines, repeating recommendations at every visit, promoting vaccination of males and females equally); and facilitating access to vaccination via community vaccination events, including back-to-school vaccination events [28,29,30].

Due to the stated barriers and identified facilitators, cancer centers have a clear role to support primary care in their consideration of vaccination rate improvement initiatives, specifically increasing HPV vaccination, in new and novel ways. The University of Texas MD Anderson Cancer Center hypothesizes that these barriers can be addressed in its service area through the intentional development of an institutional framework that is implemented via a cross-sector multi-institutional community of practice implementing evidence-based interventions. This can be achieved by building a community of practice focused on this effort and creating a mechanism to deliver resources. These intentional actions can reduce the burden on primary care professionals and create an impetus for them to address challenges to increase HPV vaccination. This also allows additional stakeholders not traditionally engaged in HPV vaccination improvement efforts, such as school districts and dental clinicians, to engage with primary care in the implementation of effective evidence-based interventions.

### 1.3. Rationale for Resource Activation

Leading health care, public health, and policy institutions across Texas have invested significantly in addressing the need for increased HPV vaccination, individually and collectively. Cancer centers are unique among this group, as they are positioned to improve the public’s health by conducting relevant research with communities, concentrating on discovery and implementation of scientific findings, and serving as champions to fuel consistent broad community adoption of evidence-based actions [31]. Recognizing an opportunity to guide aligned, multi-institutional actions respective to evidence-based intervention and catalyze near-term impact within this important public health and cancer prevention domain, The University of Texas MD Anderson Cancer Center initiated institutional actions corresponding to the President’s Cancer Panel Report [32] and the Texas Cancer Plan [33], by organizing internal teams, mobilizing resources, and defining a strategic framework focused on improving HPV vaccination rates.

MD Anderson, a National Cancer Institute (NCI)-designated Comprehensive Cancer Center, is one of the world’s most respected centers devoted to cancer patient care, research, education, and prevention. The institution defines its area of focus and influence as the State of Texas, the second most populous state in the country (29.1M residents) [34]. MD Anderson is unique among the nation’s comprehensive cancer centers in having a formal commitment to prevention as a core component of its mission for over 40 years. The institution has prioritized HPV vaccination through its Moon Shots Program™ [35]. With the creation of the Cancer Prevention and Control Platform, MD Anderson focused on accelerating the development, dissemination, and amplification of evidence-based strategies, community services, policy interventions, and knowledge targeting measurable reductions in cancer incidence and mortality at a population level. This work is led through broad visions of cancer prevention and control and community and social impact. The Cancer Prevention and Control Platform represents MD Anderson’s primary, focused activation of expertise in the science of public health practice, community-based dissemination of evidence-based practices, applied implementation science, and impact measurement for community-facing initiatives. Through a strong network of internal and external collaborators, and an explicit mandate to create and lead high-impact cancer prevention and control initiatives, MD Anderson’s leadership on the critical issue of HPV vaccination uptake was established. This paper describes the four phase approach the institution employed to develop and deploy its Institutional Strategic Framework to Increase HPV Vaccination resulting in a cross-sector multi-institutional HPV vaccination initiative.

## 2. Materials and Methods

### Establishing an Institution-Wide Effort

In 2018, MD Anderson assembled a transdisciplinary team that developed its Institutional Strategic Framework to Increase HPV Vaccination (Framework) [36], which was adopted by the institution in 2020. The Framework is consistent with MD Anderson’s formal commitment to prevention and aligns with clear opportunities in Texas to drive HPV vaccine uptake. This work is led through a matrixed leadership partnership between MD Anderson’s Cancer Prevention and Control Platform and its Office of Health Policy. With expertise from the Division of Cancer Prevention and Population Sciences, Office of Governmental Relations, and Office of the Chief Scientific Officer, the partnership oversaw the development and co-implementation of the Framework, which was created via four distinct phases in alignment with NCI Cancer Center Support Grant Community Outreach and Engagement components [37]. The Framework guides an investment in innovative strategies to increase HPV vaccination in Texas while solving for the challenges that prevent implementation of recommended strategies in a variety of settings, including traditional primary care.

In 2021, MD Anderson activated a philanthropic gift for the Cancer Prevention and Control Platform to drive innovative evidenced-based strategies to increase HPV vaccination uptake in Texas while providing capacity-building and technical assistance to support implementation of recommended strategies in primary care and community settings. To date, approximately 75% of the available funding has been invested directly in program implementation.

**Figure 1:**
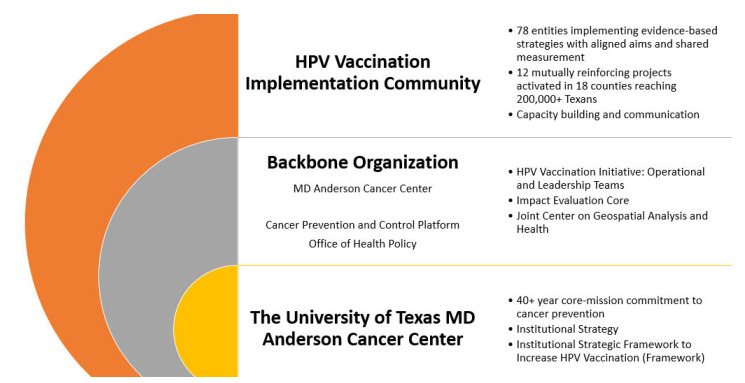
Organizational construct for the HPV Vaccination Initiative.

MD Anderson conceived of and executed the HPV Vaccination Initiative (Figure 1) through: evidence-based intervention implementation, public health practice, cross-sector partnership, systems-level change; multi-year investment, a diverse impact portfolio, a learning community, and a deep commitment to vaccine uptake as a primary objective. The institution identified initiative collaborators through data-driven outreach and constructed a portfolio of collaborative projects through an external review process designed to assess readiness, impact, and sustainability. The result is the novel HPV Vaccination Implementation Community—an ecosystem of implementation stakeholders from multiple sectors (e.g., schools, federally qualified health centers [FQHCs], academic institutions) collaboratively implementing projects within a shared measurement system.

**Figure 2:**
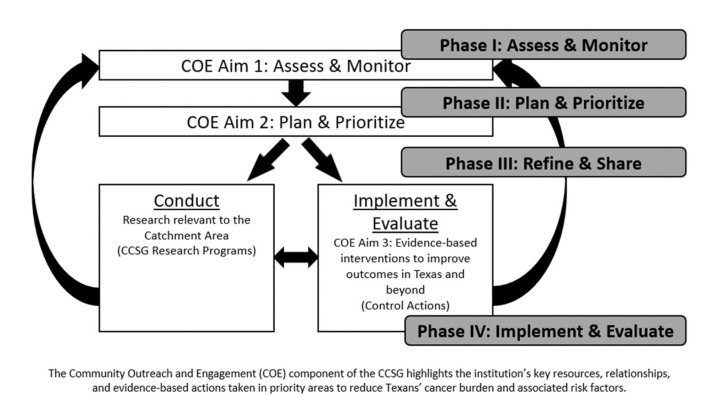
Applying the Cancer Center Support Grant (CCSG) Community Outreach and Engagement construct to create the Framework.

Creating and activating an implementation community to drive HPV vaccine uptake in Texas began for MD Anderson in September 2018 with the institution’s President commissioning definitive engagement in HPV vaccination, followed by structured convenings consisting of a multidisciplinary team of researchers, faculty, and senior/executive leaders in cancer prevention and control. The transdisciplinary team initiated development of plausible institutional approaches to HPV vaccination based upon the MD Anderson Community Outreach and Engagement Model (Figure 2). The approach consisted of four distinct phases with products designed to move from planning to project development and implementation.

Each phase was defined as follows:

Phase I: Assess and Monitor

Best practices and recommended strategies were compiled from HPV Vaccination for Cancer Prevention: Progress, Opportunities, and a Renewed Call to Action [32]; Global Routine Immunization Strategies and Practices (GRISP): a companion document to the Global Vaccine Action Plan (GVAP) [38]; CDC’s Strategic Framework for Global Immunization, 2016–2020 [39]; and 2018 Texas Cancer Plan: A Statewide Call to Action for Cancer Research, Prevention and Control [33].

Next, areas of Texas in need of intervention due to low HPV vaccination initiation and completion rates were identified. Utilizing 2020 NIS-Teen data from the CDC [5], and ImmTrac2 data requested from the Texas Department of Health and Human Service’s Immunization Registry [40], we conducted geospatial analysis to identify counties with the lowest rates of HPV vaccination. The resultant products, including static maps with layered analyses, were used as a starting point to engage potential collaborators throughout each Texas region.

Phase II: Plan and Prioritize

The HPV Vaccination Operational Team (Operational Team) selected evidence-based strategies that were consistent with best practices and strategies identified in Phase I. The team explored and defined the roles MD Anderson could uniquely assume in carrying out these evidence-based strategies. A Control Advisory Panel (CAP) was convened by the Operational Team and was composed of 39 MD Anderson faculty and staff from 21 departments, offices, and divisions. The CAP recommended that MD Anderson execute one or more roles depending on the identified need and role of external stakeholders, as well as the intervention itself and its components. The roles identified and defined by the CAP were Convener, Collaborator, Implementer, and Steward.

In each of these roles, MD Anderson generates action in one or more of the intervention categories (policy, professional and public education, community-based clinical services, and health informatics infrastructure). As Convener, MD Anderson brings partners together for common action and/or planning involving the development of a vision and strategy, creating infrastructure that supports aligned activities, facilitating the establishment of shared measures, collectively building public will and mobilizing funding. As Collaborator, MD Anderson works to bring shared resources and combined expertise to achieve shared goals. As Implementer, MD Anderson can play an active role with others in project implementation. As Steward, MD Anderson creates novel, portfolio-based implementation communities with the objectives of shared implementation and measurement, capacity building, and sustained implementation of evidence-based intervention, as structured and interwoven formally, legally and financially. This article describes how MD Anderson was activated in three of the four roles through the HPV Vaccination Initiative—as Convener, Collaborator, and Steward.

Phase III: Refine and Share

During this Phase, MD Anderson held one hour in-person meetings with 13 external expert entities and HPV vaccination stakeholders throughout Texas. During these meetings, they reviewed the set of strategies to increase HPV vaccination in Texas youth as recommended by the MD Anderson’s CAP. They validated the strategies and expressed challenges with health informatics infrastructure and requested that this be included in the final framework document. The planning core team applied this external entity feedback and added health informatics infrastructure as a separate and distinct intervention setting along with policy, professional and public education, and community-based clinical services. This addition to the intervention settings of the framework ensured a focus on strengthening capacity for electronic health record optimization, which is a critical step in the success of quality-improvement efforts across clinic systems. The final product of this extensive and thorough process was MD Anderson’s Institutional Strategic Framework to Increase HPV Vaccination (Framework), which was reviewed by MD Anderson’s President’s Advisory Council in January 2020 and fully adopted by the institution in June 2020 (after the COVID-19 pandemic delayed formal adoption of the Framework). The Framework’s four aims are:

- Aim 1: Reduce missed clinical opportunities to recommend and administer the HPV vaccine
- Aim 2: Increase parental acceptance of HPV vaccination
- Aim 3: Maximize equitable access to HPV vaccination services
- Aim 4: Strengthen the vaccination infrastructure to detect and respond to areas of need

**Figure 3:**
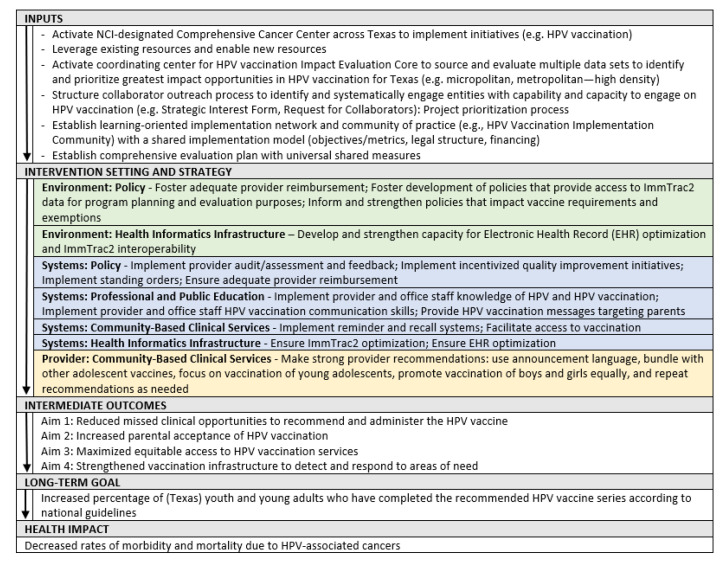
Logic model, MD Anderson HPV Vaccination Initiative.

The adopted Framework and activation of the HPV Vaccination Initiative maintains a clear logic model, which was developed based on the Implementation Research Logic Model [27] (Figure 3).

Phase IV: Implement and Evaluate

**Figure 4:**
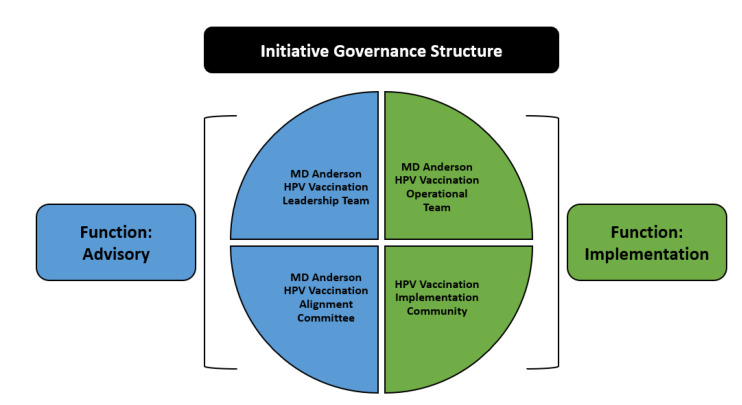
HPV Vaccination Initiative governance structure.

The Operational Team developed a governance structure to support and guide the Framework implementation and evaluation. Faculty and staff at MD Anderson utilized the Framework logic model (Figure 3) to guide the development of the strategic actions into operational plans, along with external collaborating organizations. With an established governance structure, the HPV Vaccination Initiative was established with a charge to create and activate the HPV Vaccination Implementation Community to increase HPV vaccination uptake in Texas. Further details of these efforts follow.

a. Development of a governance structure to guide the initiative. In September 2020, a four-pronged governance structure was implemented consisting of four groups: an HPV Vaccination Leadership Team, an HPV Vaccination Operational Team, an HPV Vaccination Alignment Committee, and the HPV Vaccination Implementation Community (Figure 4). The Operational Team is responsible for planning, implementation, alignment of partners/projects, and evaluation. This team meets multiple times weekly to establish and work toward achieving key milestones. The Leadership Team provides oversight through monthly meetings and small-group meetings for specific topic consensus. The HPV Vaccination Alignment Committee consists of internal experts and external reviewers/advisors.

b. Development of the co-implementation model. The MD Anderson HPV Vaccination Initiative adapted an existing co-implementation model created by the Cancer Prevention and Control Platform—Be Well Communities™ [41], which is MD Anderson’s place-based strategy for comprehensive cancer prevention and control and partnership model for community and social impact at the neighborhood level. The converted model incorporates multiple steps that engage potential collaborating organizations to ensure the collective project portfolio supports a robust and comprehensive operational plan that incorporates multiple evidence-based interventions that target regions with low vaccination rates. Based on Be Well Communities™, the Operational Team developed a Request for Collaborators (RFC) process that allowed MD Anderson, in its role as a backbone organization [42], to design shared implementation projects and activate resources to support collaborations.
c. Deployment of the co-implementation model. To implement the RFC, the Operational Team, beginning in October 2020, engaged external entities who informally advised on the development of the Framework. This resulted in a series of dialogues to discern readiness for Framework implementation and to gauge interest in formal collaborations between a range of partners across the state of Texas and MD Anderson. This targeted outreach and engagement with sharing of project portfolios, partners, and proposed plans resulted in deepening ties between organizations as well as identifying new potential collaborators. The focus on stakeholder participation enabled the Operational Team to begin formal engagement as Steward, per the logic model, to activate the novel creation of a portfolio-based implementation community. The following process is being used to develop projects in the portfolio.

The Operational Team reviewed the geospatial analyses conducted in Phase I: Assess and Monitor and identified areas of the state with the lowest adolescent HPV vaccination initiation and completion rates. The team compiled a corresponding list of HPV vaccination stakeholders from these areas that did not participate in Phase II: Refine and Share. These included, but weren’t limited to, public health units, FQHCs, Community Health Centers, hospitals, private pediatric practices, community foundations, and youth-serving community-based organizations. Each of these organizations were contacted via an email requesting an opportunity to speak with them about the initiative and the RFC. The initiative moved forward with those that participated in the previous phase and new stakeholders that were identified through this targeted outreach.

Following formational dialogues, organizations interested in formal collaboration via the HPV Vaccination Implementation Community are asked to submit a Strategic Interest Form (SIF) identifying evidence-based strategies, other potential collaborators, budget, municipal and county location(s), and current funding sources. The SIF data are analyzed and compared against the geospatial analyses conducted in Phase I (Assess and Monitor) to identify shared geographies as well as shared evidence-based interventions and cancer prevention and control domains. Organizations that submit a SIF are also invited to a web-based training on the RFC process. The Operational Team follows up with each organization after the training to discuss the proposed projects; suggest opportunities for alignment with other organizations and their respective implementation strategies, as identified through the SIF data mapping; and gauge project readiness. Focused, discerning conversations by collaborators yield a subset of organizations demonstrating project readiness and alignment with the Framework aims and strategies; these collaborators are invited to complete the RFC, which is the formal process by which engaged organizations submit competitive proposals to implement projects involving:

- Communities and infrastructure [29,43,44]
- Clinics (e.g., FQHCs, health systems, dental clinics) [45,46]
- Schools [47,48]

In addition to the assessment of overall readiness and alignment, organizations are required to meet specific eligibility requirements and adhere to the expectations of collaborating organizations, critically including commitment to collective actions that drive impact and uptake and willingness to be an active member of a shared learning community. MD Anderson also aims to optimize capacity building across several projects by minimizing start-up and program implementation time for existing projects and provides appropriate technical assistance to the initiative.

MD Anderson’s RFC process consists of four phases: (1) training webinar; (2) receipt of draft proposals; (3) review process with an opportunity for revisions; and (4) internal review of final proposals resulting in award funding. The training webinar lays the foundation for the Framework’s implementation by emphasizing MD Anderson’s roles as Convener, Collaborator, Implementer, and Steward. This approach aims to replace programmatic implementation in disconnected silos with consistent and open communication and intentional collaboration that is assessed using shared measurement. MD Anderson as the convener uses this time in the process to connect stakeholders, align organizations based on geography or technical capacity, and recommend ways to collaborate across all projects. As co-implementation proposals are matured through the collaborative process, 75% percent of all draft proposals submitted include additional partnerships and/or joint proposal development. Once the final proposal period closes, the Operational Team affirms alignment, then summarizes the collective portfolio by evidence-based strategies, geography, total partners, and overall budget. This summary prepares the proposals for the next stage in the collaboration governance model—external review and scoring. Final proposals that articulate both a problem and a sustainable solution are advanced to the next stage of review. The RFC process ensures MD Anderson collaborators address a significant barrier(s) to HPV vaccination and provide a solution that is impactful, scalable, feasible, evidence-informed, grounded in evaluation, and sustainable.

d. Creation of an External Review Panel and novel integration of scoring rubrics. The MD Anderson HPV Leadership and Operational Teams invite experts in relevant fields to serve on the External Review Panel to review and score proposals received in response to the RFC. These vetted panelists, based both in Texas and throughout the United States, represent expertise in a field activity associated with increasing HPV vaccination rates: execution of clinical quality-improvement initiatives; delivery of pediatric clinical services; implementation of multi-level public health interventions; and design and implementation of public health and/or health services research.

The Operational Team hosts the External Review Panel members for a virtual orientation and training session to provide initiative background and instructions on how to score proposals. Following the webinar, reviewers are asked to score their assigned proposals using a rubric specifically developed to assess successful implementation of evidence-based interventions. The rubric includes the following criteria: Impact, Scalability (Reach), Feasibility (Adoption & Implementation), Evidence-Informed, Evaluation (Efficacy or Effectiveness), and Budget and Sustainability (Maintenance). The rubric combines both the RE-AIM framework as well as MacArthur Foundation’s 100&Change initiative into a unique scoring design [49,50]. The overall criteria and the six domains are based on the integration of scalable solutions that advance health, achieve equity, and drive shared, aligned, sustainable impact. This process is consistent with MD Anderson’s overarching commitment to a milestone-driven environment and implementation of meaningful outcomes.

Members of the External Review Panel are matched to proposals based upon their expertise. This enables structured, overlapping subject-matter expert feedback on the proposals and assessment of their likelihoods of successful implementation and impact. Feedback and scoring from the Operational Team, Leadership Team, and External Review Panel are analyzed and applicants with favorable scores are selected for partnership. The Operational Team works with each organization to activate a formal legal and financial agreement. They work together when changes are required in the proposal and their objectives and metrics. Co-implementation partnership is predicated on shared achievement of agreed-upon milestones outlined in scopes of work.

Proposals not selected are offered the opportunity to work with MD Anderson to strengthen proposals for implementation partnerships and reapply in a future cycle. Two organizations have taken advantage of the opportunity and subsequently resubmitted proposals to begin implementation 6 months after their initial project proposal review. These best practices in governance, expert review, and stewardship ensure that individual initiatives and the overall portfolio are aligned to MD Anderson’s core values of discovery, caring, integrity, safety, and stewardship.

e. Evaluation of the HPV Vaccination Implementation Community. MD Anderson, in collaboration with its Impact Evaluation Core [51] and RTI International [52], developed a comprehensive mixed-methods evaluation plan to ensure adequate data collection and to align organizational objectives with outcomes. Established in 2019, MD Anderson’s Impact Evaluation Core aims to assess the impact of implementing cancer control initiatives in communities and with priority populations experiencing health disparities, operating on the premise that the key purpose of health program evaluation is to improve prevention, research, public health, and clinical practice. Evaluation activities occur at five levels: program impact, collective impact, shared measurement, cost-effectiveness, and community impact.

- Program impact. Program impact is an assessment of the implementation of the evidence-based interventions (EBIs). Quantitative and qualitative data on program implementation are collected from collaborating organizations via quarterly and annual reports. Each organization completes objectives and metrics tables indicating the implementation status of each EBI. These reports include procedural challenges, successes, updated metrics, and recommendations for sustainability as well as shared measures. MD Anderson reports frequencies for categorical data (e.g., the number of collaborating organizations meeting an objective) and calculates percentages as appropriate (e.g., the percentage of programs demonstrating significant change in a specific health-related outcome). Given the differences in timing of measurement and measures available to evaluate how the initiative has affected health-related outcomes among program participants, analysis is conducted to examine changes at the individual or group (e.g., class, school) level, as appropriate. For qualitative data (e.g., open-ended stakeholder survey items, stakeholder interviews), MD Anderson and RTI code for themes and interweaves data into the report to describe activities, barriers, and facilitators, and to explain trends and contextualize implementation of the programs as intended.
- Collective impact. Collective impact [53] assesses seven distinct areas that focus on the extent to which program activities were implemented as intended and improved health outcomes, as well as the collaborative impact of the initiative. The seven domains are:
  a. Implementation of Planned Program Activities/Modification of Plans
  b. Health Outcomes at Collaborating Partners and Community Levels
  c. Systems Changes at Initiative and Partner Levels
  d. Impact within the Community of Practice
  e. Lessons Learned
  f. Sustainability
  g. Cost-effectiveness

RTI International is collecting data from each implemented project via a mixed methods approach. RTI distributes the online surveys to key staff at each collaborating organization, and the surveys include a mix of closed and open-ended questions. For the interviews, RTI conducts an hour-long semi-structured interview via Zoom with each collaborating organization. Participants can discuss their programs, the HPV Vaccination Implementation Community and MD Anderson’s role in the overall initiative. Data collection from the first four projects has occurred. Data collection with additional project cycles is scheduled within 3 months of their annual report submission.

- Shared measurement. MD Anderson’s Impact Evaluation Core and RTI facilitated a discussion and sought input from collaborators to establish a set of shared measures. These measures are intended to assess the overall progress of the collaborations’ efforts. The process of developing and utilizing these common sets of measures allows the implementation community to evaluate performance and track progress toward goals. It also helps the implementation community remain aligned and accountable for intended impacts. The HPV Vaccination Implementation Community agreed upon four shared measures: (1) Rate of initiation of HPV vaccination series; (2) Completion/Up to Date HPV vaccination series; (3) Provider knowledge of HPV recommendation best practices, and (4) Provider self-efficacy for communicating with parents. Other measures that collaborators are collecting relate to electronic health records; missed clinical opportunities; and rates stratified by age, race, ethnicity, and gender; among others.
- Cost-effectiveness. As part of MD Anderson’s HPV Vaccination Initiative, RTI is conducting a cost-effectiveness analysis of each of the funded programs. Cost-effectiveness analysis compares the relative costs of achieving the same outcome by means of different activities or interventions. The analysis will be beneficial to both MD Anderson and to each of the participating organizations to help evaluate the operational sustainability of each program. Results will also inform other organizations that may want to implement similar programs. Furthermore, cost-effectiveness evaluation can assist decision-makers in allocating resources to maximize the net public health benefit when choosing among options and EBIs.

There are two crucial components to a cost-effectiveness evaluation: program costs and outcome measures. Costs are determined by an organization’s proposed budget (e.g., budgeted personnel time and supplies) and reported expenses through annual reports to MD Anderson. Expenditures considered include personnel time (e.g., time to administer vaccine, schedule appointments, talk with adolescents/young adults/parents, manage the intervention), transportation, supplies (e.g., vaccination supplies, office supplies, administrative expenses), consumables (e.g., promotional materials, printed educational materials), and in-kind expenses, as appropriate. Cost-effectiveness evaluation will be conducted by activity as feasible. Results will be presented as a cost-effectiveness ratio, and interventions will be ranked in terms of cost-effectiveness. As is feasible with the available data, considerations will also include the geographic area, target population, and/or number of potential participants in an intervention.

- Community impact. The intent is to assess the impact of participation in the HPV Vaccination Implementation Community for a collaborating organization. This includes network mapping to identify the type of engagement the organizations have with each other and whether these developed as a result of their participation. The number of grant or philanthropic proposals submitted and/or awarded that leverage the results of participation in the HPV Vaccination Implementation Community will be collected, among other community impact metrics.

## 3. Results

MD Anderson’s HPV Vaccination Initiative began by reviewing and compiling best practices and HPV vaccination rates. This was followed by convening an internal transdisciplinary team to form the Control Advisory Panel (CAP). They selected EBIs and roles that were aligned with MD Anderson’s resources and capabilities. External stakeholder feedback on the EBIs and roles was combined with that of the CAP to form the *Institutional Strategic Framework to Increase HPV Vaccination* (Framework) and a logic model to guide initiative implementation.

A governance structure (Figure 4) consisting of an operational team was put in place to support initiative implementation. A co-implementation model was developed and deployed to form the implementation community. This process was supported by an External Review Panel and is evaluated by MD Anderson’s Impact Evaluation Core and RTI, International.

**Figure 5:**
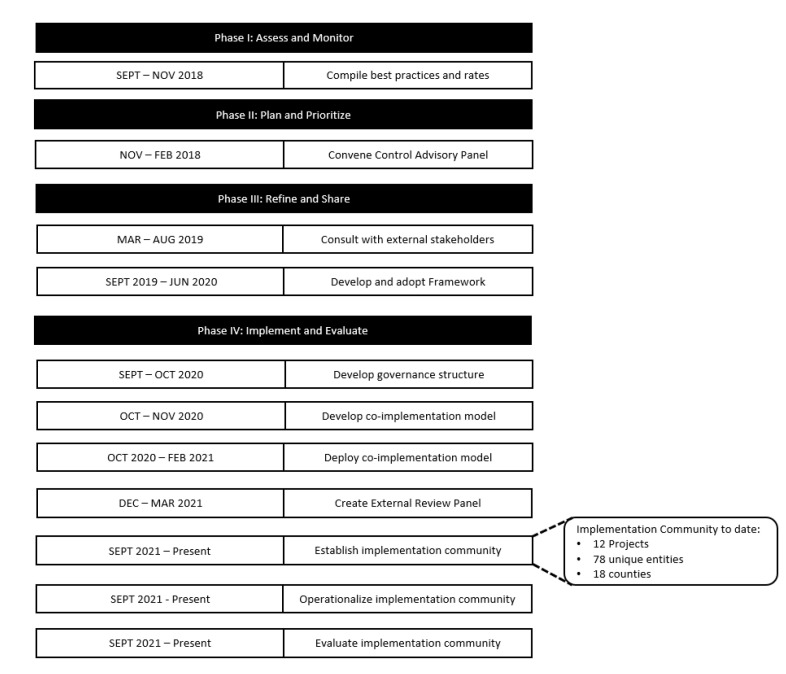
Timeline of Developing and Implementing the HPV Vaccination Initiative.

The formation of a portfolio-based HPV Vaccination Implementation Community is the result of intentional, measured actions taken by MD Anderson to define a strategic framework and activating resources to implement it to increase Texas HPV vaccination rates (Figure 5).

The current implementation community includes 12 projects that are structured and interwoven formally, legally, and financially (Table 1). They are led by three FQHC systems, one health system, six academic medical centers, one academic institution, and one academic dental center. These projects are implemented in 18 counties across five community clinical settings: community (six), FQHC systems (six), Independent School Districts (five), dental clinics (three projects), and health systems (two projects).

Projects within the overall vaccine uptake impact portfolio are implementing multilevel interventions that cover one or more of the four domains–policy, professional and public education, community-based clinical services, and health informatics infrastructure—and includes a range of five to 16 unique EBIs at the system level and/or provider level. Collectively, the HPV Vaccination Implementation Community is implementing one-hundred thirty 130 total EBIs; eighty (80) EBIs are the responsibility of the health system in which a provider engages with patients and parents, covering 10 system-level EBI types, while 50 EBIs are the responsibility of the providers themselves, covering six provider-level EBI types. Nine (75%) of the 12 projects within the vaccine-uptake impact portfolio are implementing fifty percent 50) or greater of the 16 total available EBIs. Six (50%) of the 12 projects are actively implementing EBIs in two or more implementation settings within the design of their project. This collective approach to implementation is actively reaching an estimated 200,000+ Texans, with learning and impact implications for all eligible populations. Shared measurement and comprehensive evaluations have been adopted by each individual project and the portfolio as a whole; and are intended to assess the overall progress of the collaborations’ projects.

Novel products developed by MD Anderson that supported this initiative were: conceptualization of a program impact portfolio approach to rapid learning and uptake of best practices within the context of a community of practice for vaccine health and cancer control; the Institutional Strategic Framework to Increase HPV Vaccination; the design and activation of an implementation community of practice for vaccine uptake, specifically HPV vaccine, that can be replicated for other public health priorities; an impact portfolio curation process (e.g., Request for Collaborators) and associated multi-modal scoring rubric informed by public health and philanthropic domains; a comprehensive program evaluation plan; and an agreed-upon set of shared measures to align impact measurement across various implementation settings and EBIs.

## 4. Discussion

Cancer centers are uniquely positioned to improve the public’s health by serving as entities that guide and support broad community adoption of evidence-based actions [31]. The University of Texas MD Anderson Cancer Center recognized an opportunity to assess its institutional strategy and identify a pathway to extend that strategy outward to guide and align multi-institutional actions for the implementation of evidence-based strategies to increase HPV vaccination. The four phase approach initiated institutional actions corresponding to the President’s Cancer Panel Report [32] and the Texas Cancer Plan [33], by organizing internal teams, mobilizing resources, and defining a strategic framework focused on improving HPV vaccination rates.

The COVID-19 pandemic created a challenge during the launch of Phase IV: Implement and Evaluate. Competing health care priorities, along with growing vaccine hesitancy, prevented a larger initial cohort of responders to the Request for Collaborators. Existing relationships with community partners proved beneficial in supporting early adopters. Understanding how systems changes and new community relationships could also support other community and clinical vaccination efforts also supported organizations’ decisions to engage during the pandemic.

A key component of the successful implementation of the MD Anderson strategic framework to increase HPV vaccination in Texas was not only the development of the implementation community but the detailed planning and strategy around each of the projects’ objectives and metrics. Projects were able to select the evidence-based strategies that met their needs and were not bound to a prescribed list.

These formal agreements establish not only a collective impact approach, but drive collaboration across projects and are a central factor in the success of the community of practice.

This paper describes the development of the HPV Vaccination Implementation Community based on the development of an institutional framework. The framework guided the activation of a philanthropic gift intended to drive implementation of EBIs in primary care and community settings.

As projects move further into Phase IV: Implement and Evaluate, MD Anderson will continue to support program and portfolio data collection and analysis of each project to discover the sustainability of each EBIs. Future publications will disseminate results from the individual projects, including cost-effectiveness evaluation. These data will demonstrate the time, resources, and cost-effectiveness of these interventions and their impact on communities in Texas. Future efforts will also include assessing the impact of the initiative on the community of practice and for individual members. This will include network mapping that identifies the types of linkages between implementation community projects and will speak to the ability of the initiative to achieve Aim 4 (strengthening the vaccination infrastructure to detect and respond to areas of need). Further, these learnings will be applied to and directly inform related initiatives aimed at communities of practice that address other major challenges addressable through focused health-systems strengthening.

The collaborating organizations leading the 12 projects have engaged voluntarily in this initiative. This is an overall limitation as these organizations may not be representative of all environments or entities that could disseminate evidence-based strategies within Texas.

Future directions. This specific initiative has been focused on vaccinating eligible Texas youth ages 9–17 years old. Low vaccination rates in this age range have left many young adults vulnerable to HPV infection as they transition out of pediatric care settings. A limitation of this initiative is its limited age focus. In addition, HPV vaccination data for the young adult population is scarce at the state level. “The HPV vaccination may be considered for males and females age 27–45 after a discussion with their clinician of the benefits and limitations of the vaccine for individuals in this age range. Individuals should be counseled regarding decreased effectiveness of the vaccine in those who are sexually active and already infected with one of the types of HPV in the vaccine” [54]. However, there is an opportunity to disseminate lessons learned through this initiative to target the young adult population.

## 5. Conclusions

With adoption of an institutional HPV vaccination Framework in 2020, MD Anderson established a multi-sector implementation to drive HPV vaccination uptake in Texas. The need to overcome multi-level barriers at the patient, provider, and healthcare system levels called for development of a community of collaborators from multiple sectors across Texas to align evidence-based strategies and to implement HPV vaccination in a concerted manner. Such an approach leverages the collective capacity and knowledge of collaborators in real-time during active implementation of evidence-based practices, while working together to achieve shared goals and measures.

With established public health practice infrastructure, MD Anderson will extend implementation collaborations and disseminate lessons learned to the broader health care and public health community to ensure vaccine equity–when all eligible persons have equitable access to vaccination. Through intentional initiative design, MD Anderson has prioritized building the individual and collective capacity of organizations through the HPV Vaccination Implementation Community. Notably, formal legal construct with focused goals, objectives, metrics, and milestones, set alongside key assets, such as establishing vaccine champions within their organizations, ensure focused, yet thoughtful and balanced execution of the initiative amidst myriad challenges experienced by the range of implementation settings. Without the impetus of this initiative and the paired capacity building and resources, collaborators would not have been able to so quickly and collaboratively implement quality improvement processes to increase vaccination access within their organizations nor been able to formally engage with external partners as needed, as part of an intentional, aligned community of practice.

As this initiative continues, there are key questions around sustainability to assess (e.g., sustained uptake as EBIs implementation continues; impact of policy change at the federal, state and local levels on vaccination rates). In addition, the HPV vaccination implementation community is beginning to collaborate on funding proposals that use lessons learned from this community of practice to support new implementation science and further dissemination in Texas.

Clinical healthcare practice and community based public health mediums have the potential to meet growing disparities, including providing forms of preventive care to unvaccinated adolescent populations. While this implementation network of multi-sector collaborators is already amenable to collaborating in provision of services, like vaccination, entities committed to implementation of evidence-base, including vaccination, face many real-world challenges. This implementation of a coordinated cross-sector program to provide HPV vaccinations to adolescents may be instructive to others in designing such programs. Examination of key partnership features, either contributing or detracting from overall success, will highlight future focus and refinement. An intentional approach to barrier assessment, solution building, and shared learning contributes to the overall field in public health practice.

## Figures and Tables

**Table 1:** Implementation Community by project lead entity, implementation setting, and EBI.

Implementation Setting	FQHC System 1	FQHC System 2	Health System 1	FQHC System 3	Academic Medical Center 1	Academic Medical Center 2	Academic Medical Center 3	Academic Medical Center 4	Academic Institution	Academic Medical Center 5	Academic Dental Center 1	Academic Medical Center 6	Totals for Settings and EBS
Community	⌧	⌧			⌧			⌧	⌧			⌧	**6**
FQHC System	⌧	⌧		⌧	⌧				⌧	⌧			**6**
Independent School District	⌧	⌧				⌧	⌧	⌧					**5**
Dental Clinic	⌧										⌧	⌧	**3**
Health System			⌧		⌧								**2**
Evidence-Based Interventions (EBIs)													
Implement provider & office staff HPV vaccination communications skills	●	●	●	●	●	●	●	●	●	●	●	●	**12**
Implement provider and office staff knowledge of HPV and HPV vaccination	●	●	●	●	●	●	●	●	●	●	●	●	**12**
Provide HPV vaccination messages targeting parents	●	●	●	●	●	●	●	●	●		●	●	**11**
Facilitate access to vaccination	●	●	●	●	●	●	●	●	●		●	●	**11**
Implement reminder and recall systems	●	●	●	●	●	●	●	●		●	●		**10**
Promote vaccination of boys and girls equally	■	■	■	■	■	■	■	■	■	■			**10**
Make strong provider recommendations	■	■	■	■	■	■	■		■		■		**9**
Bundle with other adolescent vaccines	■	■	■	■	■	■	■	■	■				**9**
Focus on vaccination of young adolescents	■	■	■	■	■	■	■	■	■				**9**
Repeat recommendations as needed	■	■	■	■	■	■	■						**7**
Implement provider audit/assessment and feedback	●	●	●	●	●					●		●	**7**
Ensure electronic health record optimization	●	●	●	●	●					●			**6**
Use announcement language	■	■	■	■		■	■						6
Implement standing orders	●	●	●	●						●			**5**
Ensure ImmTrac2 optimization	●	●	●	●									**4**
Implement incentivized quality-improvement initiatives	●				●								**2**
**Total Interventions by Org**	**16**	**15**	**15**	**15**	**13**	**11**	**11**	**8**	**8**	**7**	**6**	**5**	**130**

⌧ Implementation setting(s) for each project lead ● Responsibility of the system ■ Responsibility of the provider.

## Data Availability

The data presented in this study are available on request from the corresponding author, where applicable.
